# Predicting thromboembolic complications in COVID-19 ICU patients using machine learning

**Published:** 2020-10-14

**Authors:** Davy van de Sande, Michel E. van Genderen, Babette Rosman, Maren Diether, Henrik Endeman, Johannes P. C. van den Akker, Martijn Ludwig, Joost Huiskens, Diederik Gommers, Jasper van Bommel

**Affiliations:** ^1^Department of Adult Intensive Care, Erasmus University Medical Center, Rotterdam, the Netherlands; ^2^Deloitte Netherlands, Analytics and Cognitive, Amsterdam, the Netherlands; ^3^SAS Institute, Health Care Analytics, Huizen, the Netherlands

**Keywords:** COVID-19, pulmonary embolism, intensive care units, machine learning, artificial intelligence

## Abstract

**Background:**

The coronavirus disease 2019 (COVID-19) pandemic is a challenge for intensive care units (ICU) in part due to the failure to identify risks for patients early and the inability to render an accurate prognosis. Previous reports suggest a strong association between hypercoagulability and poor outcome. Factors related to hemostasis may, therefore, serve as tools to improve the management of COVID-19 patients.

**Aim:**

The purpose of this report is to develop a model to determine whether it is possible to early identify COVID-19 patients at risk for thromboembolic complications (TCs).

**Methods:**

We analyzed electronic health record data of 108 consecutive COVID-19 patients admitted to the adult ICU of the Erasmus University Medical Center between February 27 and May 20, 2020. By training a decision tree classifier on 66% of the available data, a model for the prediction of TCs was developed.

**Results:**

The median (interquartile range) age was 62 (53-70) years and 73% were male. Forty-three patients (40%) developed a TC during their ICU stay. Mortality was higher for patients in the TCs group compared to the control group (26% vs. 8%, *P*=0.03). Lactate dehydrogenase, standardized bicarbonate, albumin, and leukocytes were identified by the Decision Tree classifier as the most powerful predictors for TCs 2 days before the onset of the TC, with a sensitivity of 73% and a positive likelihood ratio of 2.7 on the test dataset.

**Conclusions:**

Clinically relevant TCs frequently occur in critically ill COVID-19 patients. These can successfully be predicted using a decision tree model. Although this model could be of special importance to aid clinical decision making, its generalizability and clinical impact should be determined in a larger population.

**Relevance for patients:**

Recently, severe TCs were observed in COVID-19 patients with progressive respiratory failure warranting ICU treatment. Timely identification of patients at risk of developing TCs is critical inasmuch as it would enable clinicians to initiate potentially salvaging therapeutic anticoagulation.

## 1. Background

A pandemic of severe acute respiratory syndrome coronavirus 2 (SARS-CoV-2) infection responsible for coronavirus disease 2019 (COVID-19) [1] has led to large numbers of moderately and severely ill patients. Up to 5% of COVID-19 patients need intensive care unit (ICU) treatment [2] due to severe pulmonary dysfunction, resulting in acute respiratory distress syndrome [3,4]. Previous reports suggest that endothelial dysfunction leads to secondary activation of the complement system and coagulation cascades, culminating in hyperinflammation, the formation of microthrombi, and corollary gas exchange disturbances and even fatal obstructive shock [5-8]. Early clinical identification of impending pulmonary issues is therefore crucial to initiate timely treatment.

Recently, severe thrombotic complications were observed in COVID-19 patients with progressive respiratory failure warranting ICU treatment [9]. Because there is a failure to identify and accurately manage this risk, the National Institute for Public Health of the Netherlands advised to lower the threshold of initiating therapeutic anticoagulation [10,11]. The clinical relevance of coagulopathy in these patients is mainly characterized by a positive correlation between elevated D-dimer levels and poor prognosis. Accordingly, therapeutic anticoagulation in high-risk individuals has yielded beneficial outcomes [12,13]. Furthermore, previous studies suggest that patients with a high probability of a pulmonary embolism could benefit from preemptive therapeutic anticoagulation, even before diagnostics based on computed tomography (CT) [14]. To date, studies regarding the early recognition of these high-risk patients are lacking. Timely identification of patients at risk of developing thromboembolic complications (TCs) is critical inasmuch as it would enable clinicians to initiate potentially salvaging therapeutic anticoagulation. Especially in a stressful time, such as the COVID-19 pandemic, diagnosis of the thromboembolic disease is difficult because few clinical CT investigations are performed either due to the overwhelming workload or due to the severity of critical illness (clinical instability). Furthermore, CT scans are often performed without iodinated intravenous contrast and very high D-dimer levels are often observed (secondary to inflammation in the later phase), complicating the interpretation [15,16].

Vast amounts of patient data are generated at a high rate during the COVID-19 pandemic and it has been difficult for clinicians to keep up and interpret these data for each individual patient. Data analysis in the form of machine learning is capable of processing large amounts of complex patient data and may contribute to making more efficient use of data and generate accurate, patient-specific predictions [17].

In the current study, we describe the number of TCs in critically ill COVID-19 patients warranting ICU treatment and provide a decision tree model to predict the risk of TCs.

## 2. Methods

This is a single-center retrospective cohort study at the adult ICU of the Erasmus University Medical Center, Rotterdam, the Netherlands. To be included, patients must have a confirmed SARS-CoV-2 infection through nasopharyngeal swab and PCR and had to be older than 18 years of age. The first patient was enrolled on February 27, 2020. Patient data were censored at the time of data cutoff on May 20, 2020. All patients had at least 14 days of hospital follow-up. All data were retrieved from the patient’s electronic health records. The study was approved by the institutional review board of Erasmus University Medical Center and the need for written informed consent was waived. All data were de-identified. The manuscript has been prepared in accordance with the transparent reporting of a multivariable prediction model for individual prognosis or diagnosis guideline for multivariable prediction models [18].

### 2.1. Predictors

As candidate predictors for the model, we considered demographic, diagnostic, and treatment data collected during ICU stay (including laboratory test results and radiologic assessments) (Table 1). Laboratory tests were performed routinely and radiologic assessments, including plain chest radiography, arterial and/or venous ultrasound, and CT pulmonary angiogram, were performed at the discretion of the treating physician [9].

**Table 1:** Predictors used in the prediction of thromboembolic complications.

Patient information (3 features)	Age, gender, BMI
Prior diseases (8 features)	Hypertension, diabetes mellitus, other cardiovascular diseases,
	ischemic stroke, tumor, chronic renal insufficiency,
	chronic lung disease, congestive heart failure
Laboratory results (32 features)	Lymphocytes, neutrophils, neutrophil/lymphocyte ratio, CRP, leukocytes, eosinophils, Il-2R, IL-6, thrombocytes, LDH, D-dimer, CKMB, hsTNT, NT-proBNP, albumin, ALAT (GPT), BSE, CK, cystatin-C, ferritin, fibrinogen, KL6, creatinine, NGAL, PCT, pO_2_, PTINR, bicarbonate, suPAR, triglyceride, APTT, APTT ratio

BMI: Body mass index, CRP: C-reactive protein, IL-2R: Interleukin-2 receptor, IL-6: Interleukin-6, LDH: Lactate dehydrogenase, hsTnT: High-sensitive troponin t, NT-proBNP: N-terminal prohormone of brain natriuretic peptide, ALAT: Alanine aminotransferase, NGAL: Neutrophil gelatinase-associated lipocalin, PCT: Procalcitonin, PTINR: Prothrombin time international normalized ratio, suPAR: Soluble urokinase-type plasminogen activator receptor, APTT: Activated partial thromboplastin time

### 2.2. Outcomes

The primary outcome was a TC. Patients were considered TC-positive (event) if they were diagnosed with pulmonary embolism established by lung CT or cardiac ultrasound (direct visualization of thromboembolism in the heart, right ventricular dilation, and/or pulmonary hypertension) or with deep vein thrombosis during hospital admission. Plain chest radiography, arterial and/or venous ultrasound, and CT pulmonary angiogram were performed at the discretion of the treating physician. Patients were considered TC-negative (reference group/non-event) if (1) radiological assessment ruled out a TC during ICU stay and/or (2) if they were discharged from the ICU during the study period and had no clinical indication for radiological assessment during ICU stay nor during follow-up.

### 2.3. Sample size

No statistical sample size calculation was performed a priori. Sample size was equal to the number of patients treated during the study period.

### 2.4. Data preprocessing

Predictors that contained ≥30% missing data were removed. We started by considering the day of diagnosis of a TC as a mark point. We analyzed all data until 5 days before this mark point for the TC-positive patients. In the TC-negative patients with radiological assessment, we selected all data from the day with the most available measurements in the period from 2 to 0 days before the radiological assessment. For the TC-negative patients who were already discharged and had no clinical indication for radiological assessment, data were collected from the day with the most available measurements in the period of day 2-day 6 of ICU stay. We chose day 6 because this was the median day of TC diagnosis. This time window was selected to ensure that the collected data would be comparable to that of the other patients included in the study. To additionally test model performance for the day of ICU admission, we also included data on the day of admission.

We chose to focus on 2 days before the mark point because this time point is clinically relevant and demonstrated good values in the preliminary analysis. When multiple measurements of the same item were available per day, we selected only the first measurement, usually occurring between 6 am and 9 am as part of the daily routine laboratory testing. BR and MD were responsible for data analysis and modeling.

### 2.5. Statistical analysis

Continuous variables are presented as the median and interquartile range (IQR). Categorical variables were reported as counts and percentages (%). To assess whether a particular predictor was significantly different between groups, we performed a Wilcoxon rank-sum test for continuous variables and a Fisher’s exact test for categorical (binary) variables. *P*<0.05 was considered statistically significant. Furthermore, the correlation between continuous variables was evaluated using Spearman’s rank correlation coefficient. Data processing and statistical analyses were performed in R 3.6.3 (R foundation) [19] and Python 3.7 (Python software foundation) [20].

### 2.6. Model development

To create the prediction model, we trained a decision tree classifier. Our choice to select a decision tree is motivated by our attempt to create an easily clinical interpretable and easy-to-use prediction model. We collected 53 predictors that encompassed several inflammation-related and coagulation-related markers (Table 1). Only predictors that exhibited a *P*-value of ≤0.05 between TC-positive and TC-negative patients were considered for modeling. We opted for this *P*-value cutoff to (1) select the patient characteristics that are more likely associated with TC and (2) to reduce the number of predictors.

To enable machine learning, patients with missing data in one of the selected predictors were removed from the dataset. The predictive model was obtained by training a decision tree classifier on a randomly selected subset of 66% out of the available data. Hyperparameters (maximum tree depth: 3, minimum samples per leaf: 3, minimum samples per split: 7, and criterion: Gini, class weights: 1 [TC-negative], 2.5 [TC-positive]) were obtained using a grid search with 5-fold repeated 5-fold cross-validation to prevent the model from overfitting to the data. The adjusted class weights achieve a higher probability of false positives compared to false negatives (missing less patients that will experience TCs). To assess generalizability to earlier days, we backtested the model after admission to the ICU and of the 5 days before the diagnosis. We assessed model performance by generating the area under the receiver operating characteristics (AUROC) using class probabilities. [21,22] In addition, we calculated the models’ sensitivity (%), specificity (%), and positive likelihood ratio. The “decision tree classifier” of the Python sklearn-library [23] was used for modeling.

## 3. Results

**Figure 1:**
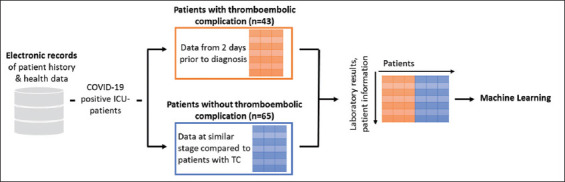
Schematic depiction of the extraction of patient data for machine learning. Data of COVID-19 positive ICU patients were extracted from the in-house database. For patients with TCs, data from 2 days before diagnosis was selected, whereas for all other patients, the data was selected to be comparable regarding the length of stay in the ICU (see section 2.4 “Data preprocessing” for more details). Finally, the predictive model was trained and tested using the resulting patient data table.

From the date of the first confirmed case up to May 20, a total of 134 patients were admitted to our ICU, of whom 108 patients were included in the analysis (Figure 1).

Demographic characteristics are presented in Table 2. The median age was 62 years (IQR, 53-70); 73% were male. Patients had a median body mass index of 28.5 (IQR, 25.9-33.0 kg/m^2^). Hypertension (38 patients; 35%) and diabetes mellitus (34 patients; 31%) were the most common chronic medical conditions. Comparing patients with TCs and those without TCs, we found no difference in the occurrence of chronic conditions. Forty-three (40%) patients developed TCs, of whom 35/43 (81%) manifested as a pulmonary embolism. Twenty-six percent of the patients with a TC died in the ICU compared to 8% in the non-TC group (*P*=0.03).

**Table 2:** Patient characteristic of patients on the day of a thromboembolic complication.

	No TCs, *n*=65	TCs, *n*=43	*P*-value
Patient information			
Age (years)	61 (46, 70)	65 (56.5, 70.5)	0.14
Sex (% male)	44 (68%)	35 (81%)	0.13
BMI (kg/m^2^)	30 (26, 33)	27 (25, 32)	0.10
Prior diseases			
Hypertension (%)	22 (34%)	16 (38%)	0.83
Diabetes mellitus (%)	18 (27%)	16 (38%)	0.62
Other cardiovascular diseases	3 (6%)	1 (2%)	0.63
Ischemic stroke	3 (6%)	0 (0%)	0.25
Tumor (hematological/solid)	9 (14%)	1 (2%)	0.07
Chronic renal insufficiency	0 (0%)	3 (8%)	0.08
Chronic lung disease	14 (22%)	9 (20%)	0.99
Congestive heart failure	10 (16%)	8 (18%)	0.99

Continuous variables are presented as median and interquartile range (25^th^ and 75^th^ percentile per group). Categorical variables were reported as counts and percentages (%). The *P*-value was calculated using Wilcoxon rank-sum test (continuous variables) or Fisher’s exact test (categorical variables), a *P*<0.05 was considered significant. TC: Thromboembolic complications, BMI: Body mass index

### 3.1. Algorithm derivation and validation

To early identify patients at risk for developing TCs, we trained a decision tree model on the available patient data. Albumin, D-dimer, leukocytes, lymphocytes, standard bicarbonate (bicarbonate), and lactate dehydrogenase (LDH) were selected (*P*<0.05) to compare TC-positive to TC-negative patients (Table 3). The neutrophil/lymphocyte ratio was excluded due to a high correlation with lymphocytes (Spearman Rank correlation >0.8).

**Table 3:** Patient characteristic on the day of a thromboembolic complication.

	No TCs, *n*=65	TCs, *n*=43	*P*-value
Patient characteristics			
CT scan (%)	25 (38%)	43 (100%)	<0.01
Pulmonary embolism (%)	0 (0%)	35 (81%)	<0.01
Venous thrombosis (%)	0 (0%)	8 (21%)	<0.01
Day of CT scan (number of days in ICU)	4 (3, 11)	7 (4.5, 10)	0.23
ICU days (until day when lab values were extracted)	4 (3, 4)	4 (2.5, 8)	0.06
Total ICU days	4 (4, 5)	5 (2.5, 8.5)	0.14
SOFA	5 (4, 7.5)	5 (5, 7)	0.47
ICU mortality (%)	4 (8%)	8 (26%)	0.03
pO_2_	10.55 (9.1, 12.65)	10.7 (9.9, 12.3)	0.42
Laboratory results			
Activated partial thromboplastin time (s)	31.5 (26.3, 46.5)	27.5 (24.5, 33)	0.19
Activated partial-thromboplastin time ratio	1.2 (1, 1.6)	1.05 (0.95, 1.33)	0.27
D-dimer, mg/L	1.205 (0.85, 2.425)	3.275 (1.7275, 4.26)	<0.01
Fibrinogen, g/L	6.8 (5.85, 7.95)	7 (5.95, 8.2)	0.49
PTINR	1.3 (1.2, 1.5)	1.2 (1.1, 1.25)	0.05
Platelets, × 10^9^/mL	276 (197, 403)	309 (261, 433)	0.13
CK, U/L	116 (50, 425)	133 (69, 248)	0.63
CKMB, U/L	1.4 (0.8, 3.5)	1.3 (0.8, 2.6)	0.57
hsTnT ng/L	18 (9, 40)	16 (10, 53)	0.58
NT-pro-BNP, pmol/L	25 (13, 62)	37 (18, 70)	0.17
Eosinophils, × 10^9^/mL	1.4 (0.575, 2.125)	1 (0.2, 1.75)	0.36
Leukocytes, × 10^9^/mL	8.2 (6.1, 10.95)	10.9 (9.25, 15.6)	<0.01
Lymphocytes, %	12.6 (8.8, 17.75)	9.5 (6.8, 12.8)	0.02
Neutrophil/lymphocyte ratio	4.7 (3.5, 8.65)	7.6 (5.25, 10.5)	0.01
Neutrophils, × 10^9^/mL	74.3 (68.93, 79.85)	79.1 (74.4, 83.1)	0.04
Serum ferritin, µg/L	992 (492, 1819)	1255.5 (787.5, 2376.25)	0.31
CRP, mg/L	190 (89, 242)	189.5 (127.5, 317.75)	0.09
IL-6, pg/L	88 (37, 125.5)	102 (64.5, 336.5)	0.07
Procalcitonin, ng/mL	0.645 (0.2, 1.895)	0.815 (0.325, 1.715)	0.31
Triglycerides, mmol/L	2.105 (1.573, 3.17)	2.23 (1.7075, 2.9925)	0.95
Creatinine, mcmol/L	76 (64, 102.75)	83.5 (74.75, 110.75)	0.16
NGAL, ng/mL	171(107, 224.25)	233 (176, 441)	<0.01
suPAR, ng/mL	12.5 (9, 20.5)	13 (11.5, 19)	0.71
ALAT (GPT), U/L	42 (26, 74)	44 (28.5, 67.5)	0.81
KL6, U/L	378 (249, 637)	548 (399, 701)	0.02
LDH, U/L	301 (247, 415.5)	355 (315.5,450.5)	<0.01
Bicarbonate, mmol/L	26.3 (23.975, 28.7)	28.5 (24.8, 31.6)	0.02
Albumin (g/L)	20 (17, 23)	16 (14, 18.75)	<0.01

Continuous variables are presented as median and interquartile range (25^th^ and 75^th^ percentile per group). Categorical variables were reported as counts and percentages (%). The *P*-value was calculated using the Wilcoxon rank-sum test (continuous variables) or Fisher’s exact test (categorical variables). A *P*<0.05 was considered significant. TC: Thromboembolic complications, CT: Computed tomography, ICU: Intensive care unit, PTINR: Prothrombin time international normalized ratio, hsTnT: High-sensitive troponin t, NT-proBNP: N-terminal prohormone of brain natriuretic peptide, CRP: C-reactive protein, NGAL: Neutrophil gelatinase-associated lipocalin, ALAT: Alanine aminotransferase, LDH: lactate dehydrogenase

Patients with missing data in one of the selected predictors were removed, yielding a final set of 76 patients for predictive modeling (33 TC-positive and 43 TC-negative). The training dataset included 50 patients (22 TC-positive and 28 TC-negative), and the testing dataset included 26 patients (11 TC-positive and 15 TC-negative).

**Figure 2:**
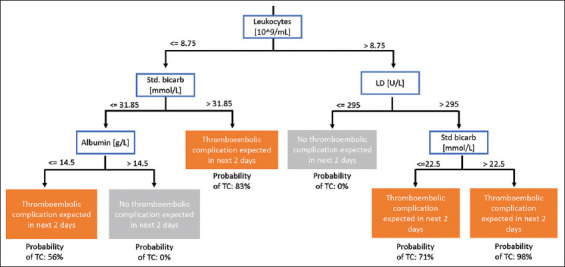
Schematic representation of the decision model to identify ICU patients at risk for TCs. By following the depicted decision rules, assessing LDH, standard bicarbonate, albumin, and leukocytes, patients that are likely to develop TCs in the next 2 days can be identified.

The strongest resulting decision model assessed the levels of LDH (U/L), leukocytes (×10^9^/L), bicarbonate (mmol/L), and albumin (g/L) (Figure 2). Five-fold cross-validation of the model on the training dataset (66% of available data) yielded an accuracy of 72% and a sensitivity of 80% on average.

### 3.2. Algorithm performance

**Figure 3:**
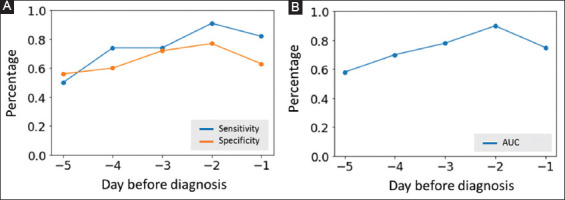
Model performance on the days before diagnosis. (A) Sensitivity and specificity of the decision model on the 5 days before diagnosis expressed in percentages. (B) AUC of the decision model on the 5 days before diagnosis.

Evaluation of this model on 33% remaining data (test dataset of 26 patients) yielded an AUROC of 0.76, a sensitivity of 73%, a specificity of 73%, and a positive likelihood ratio of 2.7. Based on these findings, we subsequently assessed whether the model could identify patients up to 5 days before TC diagnosis (Figure 3). Over time, the model demonstrated a varying sensitivity ranging from 50%, 5 days before TC, to over 90% 2 days before TC (on the total dataset). Specificity increased to 77% on 2 days before TC diagnosis.

## 4. Discussion

In this single-center case series study, we demonstrated that a machine learning-derived decision algorithm, using commonly available laboratory values, is able to predict TCs in critically ill COVID-19 patients. Model testing demonstrated high sensitivity and specificity with a positive predictive value of 67%, indicating that this decision model can improve risk stratification.

We found a high incidence of TCs in critically ill COVID-19 patients, mostly manifesting as pulmonary embolism, associated with increased mortality and as such expanding on the work of Tang *et al*. [24] and Tu *et al*. [7]. Since the global number of COVID-19 ICU patients is still increasing drastically, early detection of complications is paramount to enable appropriate and effective interventions. This study not only affirms the association between coagulation and poor outcome but also adds the clinical translation to actual prediction of such TCs.

Our model could benefit the global treatment of COVID-19 ICU patients because it is generalizable and can be reproduced in other ICU departments across the globe. Moreover, our model uses standard laboratory assessments that are easily available. Additional tests (CT-pulmonary angiography and trans-thoracic ultrasound) can be targeted to true patients at risk of developing a TC, thereby shortening the diagnostic delay and enabling early initiation of therapeutic anticoagulation therapy. However, the individual risk of bleeding must be assessed very carefully for each patient before initiating therapeutic anticoagulation.

Previous studies demonstrated that D-dimer levels were significantly elevated in COVID-19 patients admitted to the ICU with severe disease [25-27]. Increased levels of D-dimer and other fibrin degradation products have been reported in fatal cases [24] and it has even been suggested that therapy that blunts the increase in D-dimer levels might improve outcome [28,29]. In line with these observations, the National Institute for Public Health of the Netherlands advised to lower the threshold of initiation of therapeutic anticoagulation. However, this is not without possible harm because of the side effects of systemic anticoagulation and consequent bleeding. Poisey *et al.*, therefore, recently advised to be more reticent [11]. Although D-dimer was a strong predictor of TCs and was significant difference between groups, this value was not selected by machine learning. In preliminary analysis, a model including D-dimer was created but revealed no superior accuracy, sensitivity, and specificity over the current model. In addition, D-dimer contained 10% more missing data than the other variables considered for modeling and thus reduced the number of patients that could be included in the study. Our model instead selected LDH, leukocytes, bicarbonate, and albumin. LDH is an intracellular enzyme found in lung tissue (isozyme 3) and can be released to the circulation in severe infections as part of cytokine-mediated tissue damage. Correspondingly, it makes sense from a pathophysiological point of view that LDH is released in high-risk COVID-19 patients. Earlier studies demonstrated that LDH levels can be used as a hallmark of disease in in patients with MERS and COVID-19 [30]. Metabolic acidosis, which is influenced by bicarbonate, is not a benign condition and signifies an underlying disorder that needs to be corrected to improve outcomes. Metabolic acidosis was found to be markedly lower in deceased COVID-19 patients than in recovered patients [15].

ICUs provide a highly challenging environment for healthcare workers. Continuous routine monitoring in combination with repeated diagnostic assessments provides large amounts of data throughout the day. Rapid and appropriate decision making is therefore required to increase diagnostics throughput and to avoid a delay in treatment. Machine learning algorithms can provide decision support by uncovering hidden clinically relevant patterns. By using routinely collected data, our method can easily be translated to other clinics. Given their routine adoption in clinical chemistry and role in COVID-19-related pulmonary pathophysiology, it makes perfect sense that our model selected LDH, leukocytes, bicarbonate, and albumin as the strongest predictors of our model. Applying our model to all data up to 5 days before diagnosis revealed a sensitivity ≥74% already starting on day 4 before a diagnosis of a TC, which is a powerful statistic despite our small sample size.

This study has several limitations. First, not all patients in the TC-negative group underwent radiological assessment (*n*=25, 38%). The indication for additional radiological assessment was based on the decision of the attending physician in the wake of lacking international guidelines [10]. Second, we used a decision tree model to identify the early predictors of TCs. Decision trees are one of the many machine learning options to make use of the available data. In our preliminary analysis, we also considered and shortly investigated other algorithms such as logistic regression and neural networks. We selected a decision tree classifier over other algorithms since the decision tree is easier to interpret and implement in clinical settings than the output of, for example, a logistic regression [31]. Third, the small size of the validation cohort might limit the reliability of our model. External validation in a larger or prospective dataset could further address this limitation but is not yet possible as we are currently still building a national registry in the Netherlands (https://covidpredict.org) to perform more comprehensive data analysis.

## 5. Conclusions

In this single-center case series study in COVID-19 patients admitted to the ICU, we demonstrated a high incidence of TCs associated with increased mortality. A simple and generalizable decision tree demonstrated a powerful model to early recognize patients at risk for TCs. This model uses conventional clinical biochemical tests and as such represents an easy stratification tool. Further research should focus on improving model and classification performance, evaluating its performance, generalizability, and clinical impact in a larger ICU patient population.

### Conflicts of Interest

The authors declare that they have no conflicts of interest.
